# Post-operative cerebral oximetry for detection of low-cardiac output syndrome after coronary artery bypass surgery

**DOI:** 10.1007/s10877-025-01359-y

**Published:** 2025-09-13

**Authors:** Miriam Silaschi, Jacqueline Kruse, Maria Wittmann, Itsuki Osawa, Tadahiro Goto, Markus Velten, Marcus Thudium, Marc Rohner, Marwan Hamiko, David Rowlands, Stefan Kreyer, Marc Coburn, Farhad Bakhtiary

**Affiliations:** 1 Department of Cardiac Surgery, Heart Center Bonn, University Hospital Bonn, Bonn, Germany; 2 Department of Anesthesiology and Intensvie Care Medicine, University Hospital Bonn, Bonn, Germany; 3 Department of Emergency and Critical Care Medicine, The University of Tokyo Hospital, Tokyo, Japan; 4 Division of Cardiology, Department of Medicine, Columbia University Irving Medical Center, New York, NY USA; 5 TXP Medical Co. Ltd., Tokyo, Japan; 6 Department of Cardiac Anesthesiology, University of Texas, Southwestern Medical Center, Dallas, USA

**Keywords:** Low cardiac output syndrome, Hemodynamic monitoring, Cerebral oximetry, Cerebral near infrared spectroscopy

## Abstract

While cerebral near infrared spectroscopy (NIRS) is a valuable diagnostic tool to monitor brain oxygenation during cardiac surgery, its value in low cardiac output syndrome (LCOS) in adults has not been evaluated. This study was prospective and observational. Patients undergoing coronary artery bypass grafting (CABG) with reduced ejection fraction (LVEF < 35%) were included and received NIRS monitoring for up to 48 h after surgery with simultaneous continuous cardiac index (CI) monitoring. The primary endpoint was LCOS by a standard definition. From 2020 to 2023, 82 Patients with severely reduced LVEF undergoing CABG were included. Of these, 44 patients had sufficient NIRS and CI data for further analyses. Median age was 68 years (Interquartile range (IQR) 60–73), 91% (40/44) were male and median EuroSCORE II was 3.2% (1.7–5.4). Median LVEF was 30% (26.5–30.1) and baseline CI was 2.15 L/min/m² (2.05–2.60). CABG was combined with other procedures in 23% (10/44). LCOS rate was 11% (5/44) and in-hospital mortality was 2.2% (1/44). The performance of Lasso-regularized models increased if NIRS was included in LCOS prediction models (AUROC 0.99 [95%CI, 0.98-1.00]) showing that both relative NIRS drop rate and absolute NIRS value were significant predictors of LCOS. Risk of LCOS was high if NIRS drops by > 20% or absolute NIRS drops below < 50. In patients with LCOS, NIRS drop occurred before CI values decreased. NIRS drop was not associated with other adverse events. NIRS is an early and valid indicator of LCOS in patients after cardiac surgery. In selected patients, NIRS may be a substitute for invasive continuous CI measurements. However, we could not show an association of NIRS drop with adverse events. Future studies should compare blinded and non-blinded NIRS monitoring to investigate possible impact on clinical outcomes further.

## Introduction

Cerebral oximetry is a non-invasive tool to monitor regional oxygen saturation of the frontal cortex. Using basic physical principles of different absorption and dispersion of infrared light, oxygenated and deoxygenated hemoglobin can be distinguished. Thus, cerebral oximetry estimates oxygenation of blood approximately 25 mm behind the skull [1]. It represents mostly venous oxygenation of blood, reflecting balance or imbalance of oxygen supply and demand. However, any given value by cerebral near infrared spectroscopy (NIRS) is mainly a trend value, dependent on its individual baseline. The average baseline for cardiac surgical patients is 62%, ranging from 56 to 67% [1]– [2]. Also, there are various covariates influencing cerebral oxygenation, such as arterial blood oxygenation, concentration of hemoglobin, partial pressure of carbon dioxide, mean arterial pressure, depth of sedation and body temperature. Nevertheless, cerebral oxygenation has a close relation to cardiopulmonary function, as systemic supply of oxygen is also depending on cardiac output [1]. Any reduction in cardiac output, due to systolic failure or venous congestion, may lead to reduced cerebral oxygenation. Currently, intra-operative use of NIRS during cardiac surgery is recommended by German guidelines to detect cerebral desaturation early and improve neurological outcome of patients. Guidelines also acknowledge the close correlation of cerebral oxygenation and central-venous/mixed-venous oxygenation [2]– [3]. Yet, there is no recommendation or evidence base for routine use of NIRS in the postoperative course on intensive-care-units (ICU) after cardiac surgery in adult patients [2]. In theory, NIRS may limit the gap in early recognition of potential life-threatening complications present on cardiac ICU in situations of low cardiac output. This was suggested by three case series [4–6], where in situations of pericardial tamponade with consecutive low cardiac output a drop in NIRS was the earliest indicator of this critical complication. In infants with congenital cardiac disease, NIRS has been shown to be a strong predictor of low cardiac output syndrome (LCOS) [7].

However, to date no prospective study investigates the use of NIRS on ICU after adult cardiac surgery with simultaneous continuous cardiac index monitoring to investigate its alteration during LCOS. There is evidence that LCOS may occur in 20% of chronic heart failure patients after coronary artery bypass grafting (CABG) [8] and diagnosis is often delayed until clinical signs become obvious. Also, no threshold values for pathological changes in LCOS have been defined, while the general assumption is that any fall below 50% is pathologic.

The aim of this study is to investigate the diagnostic accuracy of NIRS for LCOS.

## Patients and methods

### General methods

The study was performed at the University Hospital Bonn in Germany. The target condition tested was low cardiac output syndrome (LCOS), with cerebral NIRS as the index test and Swan Ganz catheter measurements with continuous cardiac index monitoring as reference standard. For NIRS measurement, INVOS™ 5100 C produced by Medtronic Inc. (Minneapolis, MN, USA) were used, allowing for data transfer of continuous measurements on a USB portable device and data analysis on a dedicated software (INVOS™ Monitoring System Analytics Tool). For continuous cardiac index monitoring, HemoSphere Advanced Monitor (Edwards LifeScirences Inc., Irvine, Calif, USA) was used in all patients.

### Inclusion and exclusion criteria

Inclusion and exclusion criteria were described in detail previously [10]. In summary, patients undergoing coronary artery bypass surgery (either isolated or as combined procedure) with pre-operative left ventricular ejection fraction (LVEF) of < 35% according to echocardiography or angiography were candidates for inclusion. When patients were hemodynamically stable upon admission to ICU (max. dose epinephrine ≤ 0.05 µg/kg/min, dobutamine ≤ 3 µg/kg/min, enoximone/milrinone ≤ 2 µg/kg/min, Noradrenaline ≤ 0.2 µg/kg/min, Base excess ≥ −10, pH > 7.30 and Cardiac index ≥ 80% of the pre-operative value) and not on mechanical circulatory support, they qualified for further NIRS and CI monitoring. 82 patients were consented for this study between 2020 and 2023. From these, 2 patients with LVAD were excluded, 5 patients with extracorporal membrane oxygenation (ECMO), 1 patient with intra-aortic balloon pump (IABP) and 3 patients already had manifest LCOS upon ICU admission. From the remaining patients, 44 patients had sufficient continuous cardiac index (CI) and NIRS data for further analysis. Sufficient in this context means at least 18 h of continuous calibrated CI and NIRS measurements, in case of displaced electrodes, the electrodes were substituted with new electrodes and a gap no longer than hour was accepted.

### Study visits

At induction of anesthesia, baseline values for NIRS, Swan-Ganz catheter measurements, blood gas analysis and standard hemodynamic measurements were obtained. Upon admission to ICU, screening was repeated, and patients were excluded or further monitored according to inclusion/exclusion criteria. NIRS and CI was continued for up to 48 h post-procedure, if clinically indicated, and could be terminated beforehand if patients left the ICU. 30-day survival was assessed via telephone survey.

### Primary endpoint

The primary endpoint was LCOS and its timepoint of onset. Diagnosis of LCOS was based on the definition given by the study protocol [10]. LCOS was defined as the occurrence of the following conditions:

1) Two consecutive measurements of low cardiac output (defined as a cardiac output of ≤ 2.2 L/min/m^2^, without associated relative hypovolaemia) after calibration OR.

2) One measurement of low cardiac output ≤ 2.2 L/min/m^2^ plus the use of two or more inotropes OR.

3) If baseline cardiac index was ≤ 2.2 L/min/m^2^, then any measurement showing a reduction of > 20% of its pre-operative value were counted as LCOS.

The follow-up period for LCOS onset was 48 h from ICU admission or until earlier discharge from the ICU within 48 h.

### Data management

Data was collected using RedCap (Vanderbilt University, Nashville, Tennessee, USA). NIRS Data was obtained on a portable device and analyzed on a dedicated software (INVOS™ Monitoring System Analytics Tool) and ultimately exported to Microsoft Excel. Data was collected and is reported in accordance with the STARD criteria [9].

### Clinical parameters

We used the following clinical parameters at ICU admission to predict future LCOS: patient demographics (i.e., age, sex, and body mass index (BMI)), vital signs (i.e., MAP), blood gas parameters (i.e., pH, PaO2/FiO2 ratio, and base excess), laboratory data (i.e., hemoglobin and lactate), and other clinical parameters (i.e., SvO2, ScvO2, SVR, CI, and central venous pressure (CVP)). In addition, we selected three NIRS-related parameters: NIRS at ICU admission, the maximum percentage of NIRS drop from baseline at ICU admission during the follow-up period, and the minimum NIRS value during the follow-up period.

### Sample size considerations

The sample size was estimated using the lasso-regularized logistic regression model with the following hypothesis: to achieve a c-statistic of 0.95 using only three predictors (i.e., assuming that the coefficients of the remaining 11 predictors are reduced to zero) with our primary outcome (i.e., LCOS) prevalence of 0.1. The result of this estimation process indicated that the ideal sample size was 139. However, the prevalence of patients with severely reduced LVEF undergoing CABG was unexpectedly low, and enrolling 139 patients was not feasible. Therefore, we also used the 10-fold cross-validation method to minimize potential overfitting in our datasets of 44 patients.

### Statistical methods and analysis

We used summary statistics to delineate patient characteristics and operative details (Tables 1 and 2). Continuous values are given with median and interquartile range unless stated otherwise.

**Table 1:** Patient characteristics

Characteristics	*N* = 44
**Patient demographics**	
Age	68 (60, 73)
Male sex (%)	40 (91)
BMI	29.1 (25.1, 31.2)
**Preoperative information**	
EuroSCORE II	3.2 (1.7, 5.4)
STS Risk Score	1.2 (0.57, 2.6)
NYHA Classification	3 (2, 3)
**Comorbidities**	
Previous cardiac surgery (%)	0 (0)
Previous cardiac intervention (%)	12 (27)
Stroke (%)	4 (9.1)
ESRD requiring dialysis (%)	1 (2.3)
**Laboratory data**	
Hemoglobin [g/dL]	13.9 (13.1, 15.0)
Lactate [mmol/L]	1.47 (0.93, 1.86)
**Other clinical parameters**	
LV aneurysm (%)	4 (9.1)
LVEF [%]	30.0 (26.5, 30.1)
SVR [dynes/seconds/cm^−5^]	1 068 (794, 1290)
TAPSE [mm]	21.0 (17.0, 23.0)
LVEDD [mm]	5.30 (4.80, 5.80)
CI [L/min/m^2^]	2.15 (2.05, 2.60)
CVP [mmHg]	9.5 (7.0, 11.0)

Continous values are given with median and interquartile range. Abbreviations: BMI: Body mass index, STS: Society of Thoracic Surgeons, NYHA: New York Heart Association class, ESRD: end stage renal disease, LV: left ventricle, LVEF: left ventricular ejection fraction, SVR: systemic vascular resistance, TAPSE: tricuspid annular plane systolic excursion, LVEDD: left ventricular end diastolic diameter, CI: cardiac index, CVP: central venous pressure

**Table 2:** Intra- and postoperative outcomes

Characteristics	*N* = 44
**Operative information**	
Surgery type	
Elective (%)	41 (93)
Urgent/Emergent (%)	3 (6.8)
Combined procedure (%)	10 (23)
Aortic valve (%)	6 (14)
Mitral valve (%)	3 (6.8)
Ascending aorta (%)	1 (2.3)
Procedure duration [mins]	225 (175, 269)
Cradioplegic arrest used (%)	17 (38.6)
**ICU admission information**	
**Vital signs**	
MAP [mmHg]	72 (66, 80)
**Blood gas**	
pH	7.33 (7.29, 7.38)
PaO2/FiO2 ratio	234 (185, 291)
Base excess	1.48 (1.19, 1.85)
**Laboratory data**	
Hemoglobin [g/dL]	12.1 (10.5, 13.0)
Lactate [mmol/L]	1.47 (0.93, 1.86)
**Other clinical parameters**	
SvO_2_	72 (67, 76)
ScvO_2_	71 (65, 76)
SVR [dynes/seconds/cm^−5^]	753 (661, 876)
CI [L/min/m^2^]	3.10 (2.70, 3.40)
CVP [mmHg]	9.0 (6.0, 12.7)
NIRS	72 (64, 75)
**NIRS during the follow-up period**	
Minimum NIRS value	58 (53, 64)
Maximum % of NIRS drop [%]	17 (12, 24)
**Outcomes**	
LCOS (%)	5 (11)
In-hospital mortality (%)	1 (2.2)
ICU stay duration [hours] (*N* = 43)	49 (24, 106)
Hospital stay duration [days]	14 (10, 18)

Continuous values are given with median and interquartile range. Abbreviations: ICU: Intensive care unit, MAP: mean arterial pressure, FiO2/paO2: inspiratory fraction of oxygen, SvO2: central venous oxygenation, SVR: systemic vascular resistance, CI: cardiac index, CVP: central venous pressure, NIRS: near infrared spectroscopy, LCOS: low cardiac output syndrome

**Fig. 1:**
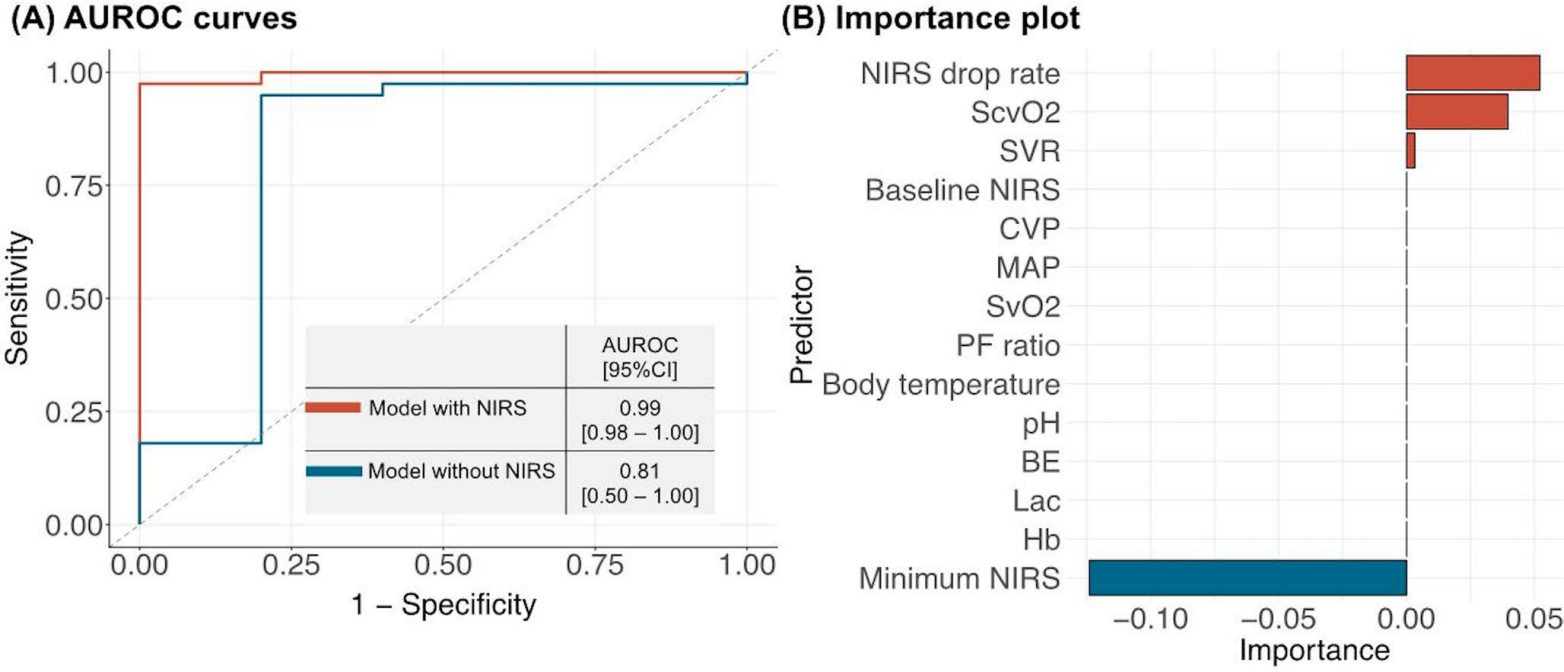
(**A**) AUROC curves and (**B**) predictor importance plot

To identify determinants of predicting LCOS, we developed two Lasso-regularized logistic regressions (Lasso regression) using (1) all candidate predictors with three NIRS-related parameters and (2) candidate predictors without NIRS-related parameters. Lasso regularization extends the standard regression model by incorporating a regularization parameter (lambda) to shrink the beta coefficients toward zero, thereby minimizing potential overfitting in the model [11]– [12]. We used several techniques to minimize potential overfitting, such as lasso regularization and 10-fold cross-validation. The predictive performance of each model is shown using AUROC curves with c-statistics in Fig. 1A.

**Fig. 2:**
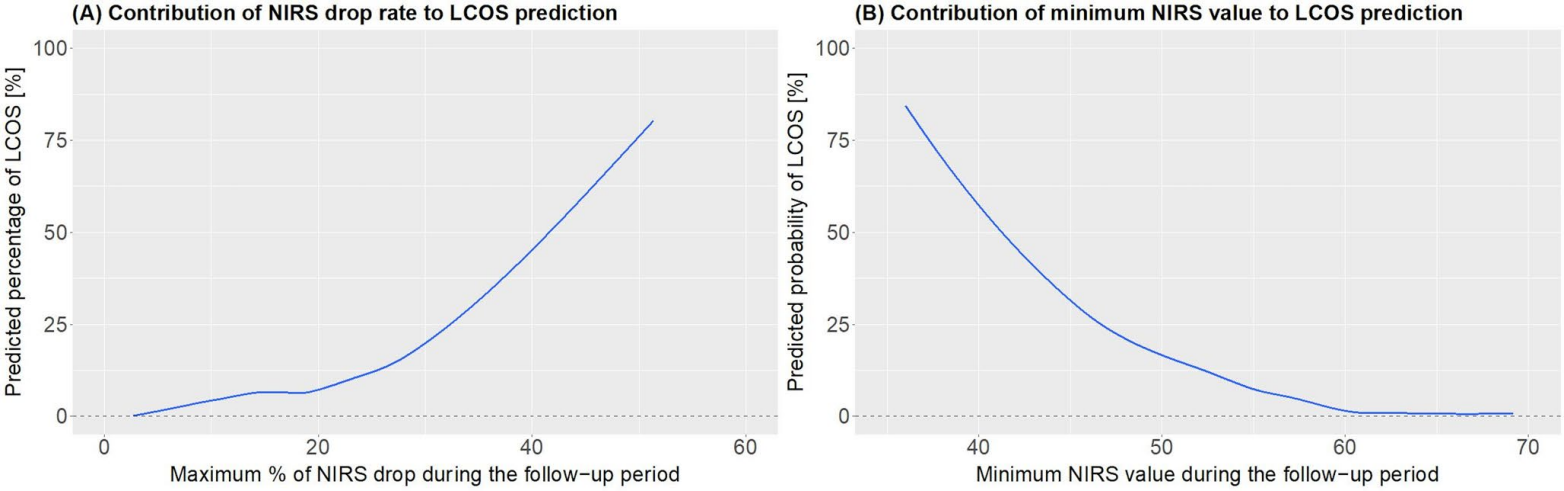
Contribution of NIRS to LCOS prediction during the study period. (**A**) maximum percentage of NIRS drop from baseline at ICU admission; (**B**) the minimum NIRS value during the follow-up period

**Fig. 3:**
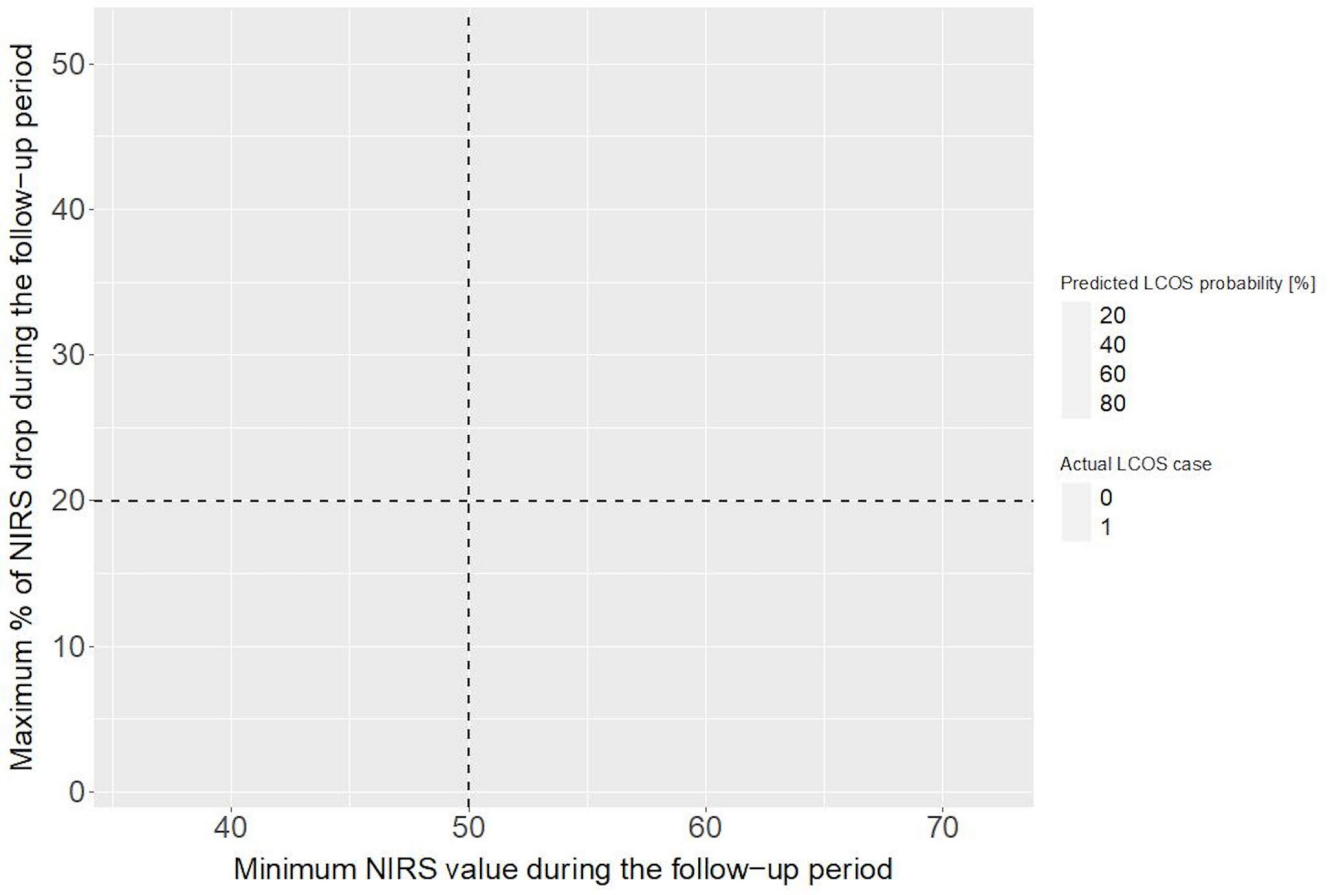
Association of NIRS drop rate and minimum NIRS value with LCOS

We reported the predictor importance, which reflects the coefficients of each prediction, in our lasso regularization model with NIRS-related parameters (Fig. 1B). Parameters with high importance, regardless of positive or negative values, were the determinant predictors of LCOS. To determine the optimal threshold of two NIRS-related parameters (i.e., the maximum percentage of NIRS drop from baseline at ICU admission and the minimum NIRS value) during the follow-up period in predicting LCOS, we visualized the contribution of each NIRS-related parameter to LCOS prediction during the study period (Fig. 2). We also showed the association of absolute NIRS value and NIRS drop rate with LCOS (Fig. 3).

**Fig. 4:**
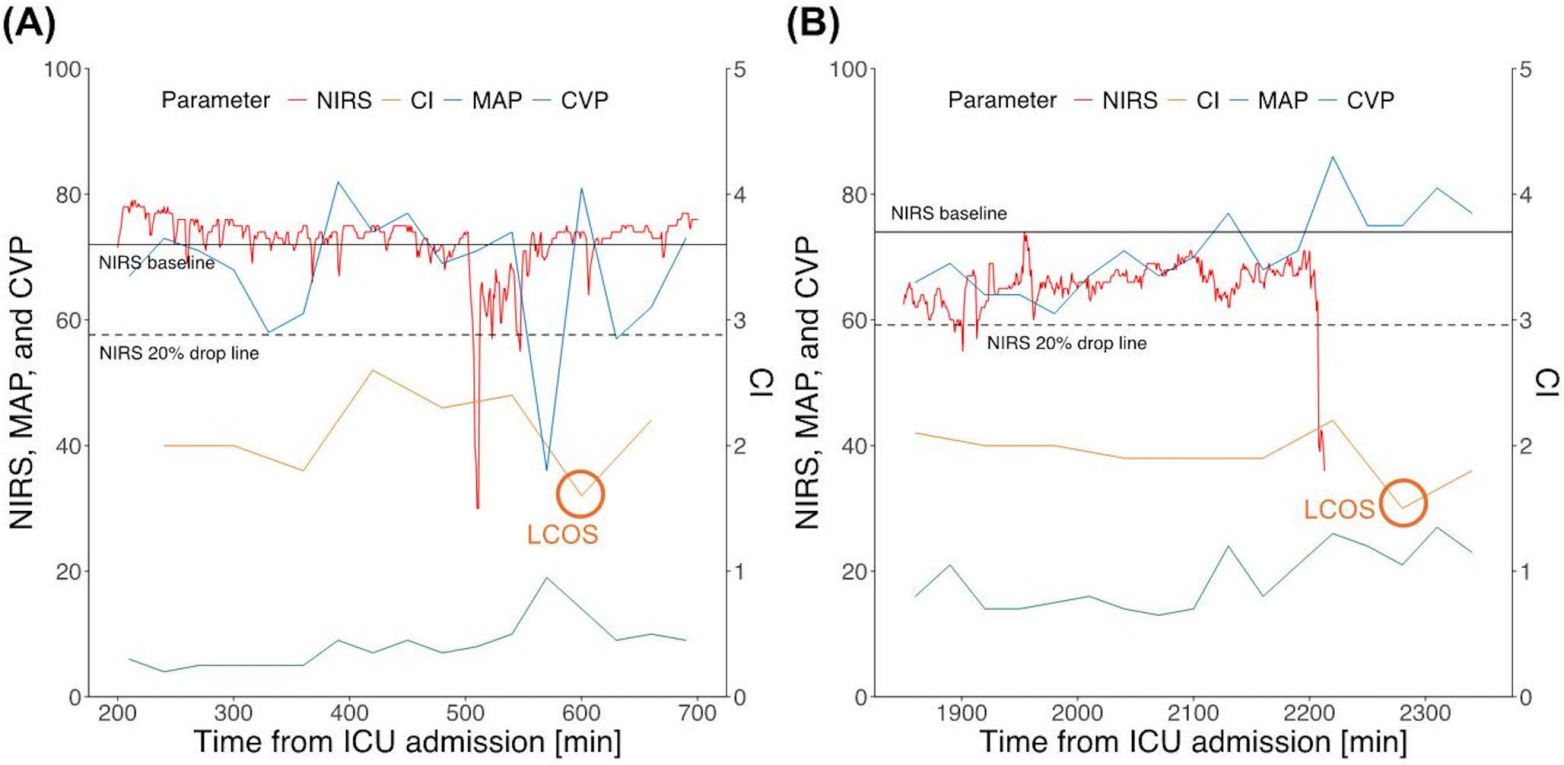
Trend of NIRS and other clinical parameters before onset of LCOS

Finally, we examined the trend of NIRS in patients with LCOS by comparing it visually with other clinical parameters such as mean arterial pressure (MAP) and CVP, which can be easily and continuously monitored in the ICU setting (Fig. 4).

A p-value of < 0.05 was considered statistically significant. All analyses were performed using R version 4.3.1. (The R Foundation for Statistical Computing).

## Results

From 2020 to 2023, 82 Patients with severely reduced LVEF undergoing CABG were included. From these, 44 patients had sufficient NIRS and CI data for further analyses. Median age was 68 years (IQR 60–73), 91% (40/44) were male and median EuroSCORE II was 3.2% (1.7–5.4, see Table 1). Median LVEF was 30% (26.5–30.1) and baseline CI was 2.15 L/min/m² (2.05–2.60). CABG was combined with other procedures in 23% (10/44). LCOS rate was 11% (5/44) and in-hospital mortality was 2.2% (1/44, see Table 2). The performance of Lasso-regularized models increased if NIRS was included in LCOS prediction models (AUROC 0.99 [95%CI, 0.98-1.00]) showing that both relative NIRS drop rate and absolute low NIRS value were significant predictors of LCOS (Fig. 1). Potential criteria for high suspicion of LCOS were statistically determined as NIRS drops by > 20% or the absolute NIRS value drops below < 50 (Figs. 2 and 3). In patients with LCOS, NIRS drop occurred before CI values decreased below the threshold of 2 L/min/m² (Fig. 4).

### Association with adverse events

There was not association of NIRS drop (drop below 50 or > 20% of baseline) with early postoperative mortality (0% in non-NIRS drop patients vs. 5.8% in NIRS drop patients, *p* = 0.4), delirium (11% non-NIRS drop vs. 12% NIRS drop patients, *p* > 0.9), renal failure (11.1% in non-NIRS drop vs. 12% in NIRS drop, *p* > 0.9), hours to extubation (10 h in non-NIRS drop vs. 13 h in NIRS drop, *p* = 0.7) or postoperative maximum troponin values (*p* = 0.6).

Median ICU stay was numerically longer in patients with NIRS drop (82 h vs. 43 h) but this did not reach statistical significance (*p* = 0.2).

### Comment

We present a study for further evaluation of the role of cerebral venous oxygenation during LCOS and hypothesized that NIRS could be used for prediction of LCOS in adult patients on intensive care unit after cardiac surgery. Using simultaneous monitoring of continuous cardiac index and NIRS, we saw that NIRS is a strong predictor of LCOS even after adjustment for possible confounders and covariates. A NIRS drop by > 20% or below a value of 50 should be considered a relevant indicator. Also, NIRS drop in LCOS patients occurred before alterations in CI were observed. This was also true for other hemodynamic parameters, such as mean arterial pressure. However, there was no association of NIRS drop with adverse clinical outcomes, but our cohort was underpowered by a limited number of patients and events to show an effect with regard to clinical outcomes. The main hypothesis of the study was, however, confirmed by our observations.

Patients undergoing cardiac surgery present a valuable cohort for analysis of a clinical scenario such as LCOS, since those with pre-operative severely reduced left ventricular ejection fraction are at specific risk to develop some form of LCOS in the early postoperative course. Current guideline recommendations favour invasive hemodynamic monitoring in those patients on cardiac ICUs. However, due to possible complications such as arrhythmia and possible technical challenges in placing the catheter, guidelines are not always followed and hemodynamic monitoring is often established at a later point, when impaired hemodynamics occur [2]. Moreover, methods like arterial pulse contour analysis or repeated transesophageal echocardiography to assess cardiac functions are still invasive and in case of transesophageal echocardiography, more advanced medical skills are necessary. This study gathered evidence that continuous NIRS monitoring may be advantageous as a non-invasive technique detecting LCOS during the early post-operative course. Possibly, NIRS monitoring could act as a step on the diagnostic ladder where more invasive forms of hemodynamic monitoring and diagnostics are established once decreased NIRS values are seen. To date, this is the only study to simultaneously correlate continuous cardiac index and continuous NIRS through a 48 h postoperative period in adult cardiac surgical patients.

Three publications already hinted at the significance of NIRS monitoring in LCOS through case reports and case series [4–6]. The value of NIRS in the setting of pediatric congenital cardiac patients has been studied more thoroughly [7, 13]. Doctor et al. studied cerebral NIRS in single-ventricle infants after stage 1 palliation and found out, that in case of low cardiac output, a NIRS drop below 57% had a sensitivity of 91% for this clinical scenario. However, as in our cohort, NIRS drop was not associated with adverse outcomes [7]. Janaillac et al. studied the correlation of cerebral NIRS and LCOS in extremely preterm infants in the first 72 h of life and found a strong correlation between NIRS values and cardiac output in these patients, where incidence of LCOS was frequent [13].

In adult patients, an association of low baseline NIRS values with chronically impaired LVEF and echocardiographic signs of congestion has also been demonstrated [14]. Although this speaks for an association of low baseline NIRS values in situations of chronically impaired LVEF, it emphasizes that cerebral oximetry is closely connected to cardiopulmonary function and states of congestion. There are various methods to assess cardiopulmonary function in the intensive care setting, however, NIRS is non-invasive and easily assessed, and may therefore be used instead of continuous CI measurements in selected patients or rather beforehand.

Since NIRS measurements were not blinded, decreased values themselves may have led to further diagnostics and clinical decision-making. This is possibly why we did not see any obvious differences in clinical outcomes, since decreased NIRS values themselves may have provoked further action. In our cohort we found that ICU stay was longer in patients with relevant NIRS drop, but this was only an obvious clinically-meaningful difference without statistical significance.

As there were very few fatalities in our cohort, our study was not powered to assess association of NIRS drop with early postoperative mortality or other clinically relevant adverse events. A blinded study with a larger cohort of patients would be more suitable to investigate impact of continuous postoperative NIRS monitoring on clinical outcomes further.

### Limitation

A major potential statistical limitation is the statistical model overfitting. We acknowledge that the potential inadequacy of the sample size could be a source of optimism (i.e., overestimated c-statistics) even in the lasso-regularized logistic regression model. Since the number of patients with LCOS is relatively small, our model may be overfitted in our datasets. However, we tried to minimize this issue by using lasso regularization and 10-fold cross-validation. Furthermore, the aim of this study is not to develop a prediction model of LCOS in the ICU setting but to investigate the clinical utility of NIRS as an early warning parameter of LCOS. Therefore, this potential limitation would not affect our conclusions regarding the potential utility of NIRS.

The number of drop outs due to either necessity for extracorporeal mechanical support before admission to ICU or insufficient measurements for analysis was higher than expected, therefore, from 82 included individuals, only 44 patients were available for extensive further analyses, therefore the study was not powered to investigate impact of NIRS on other clinically relevant adverse events (mortality, ICU stay, renal failure etc.). Insufficient measurements resulted from either missing calibration of Swan Ganz catheters, extraction due to suspected arrhythmia, removal of NIRS electrodes due to perspiration or patient discomfort or early discharge from ICU due to hemodynamic stability with fast track transferral to intermediate care units.

## Conclusion

NIRS is an early and valid indicator of LCOS in patients after cardiac surgery. In selected patients, NIRS may be a substitute for invasive continuous CI measurements. However, we could not show an association of NIRS drop with adverse events. Future studies should compare blinded and non-blinded NIRS monitoring to investigate possible impact on clinical outcomes further.

## Data Availability

Data can be made available publicly upon reasonable request.

## Abbreviations

ACS: Acute coronary syndrome
CABG: Coronary Artery Bypass Grafting
CVP: Central venous pressure
CI: Cardiac index
ECMO: Extracorporal membrane oxygenation
FiO2: Fractional concentration of oxygen during inspiration
IABP: Intra-aortic balloon pump
ICU: Intensive Care Unit
LCOS: Low Cardiac Output
LVEF: Left ventricular ejection fraction
MAD: Mean arterial pressure
NIRS: Cerebral near infrared spectroscopy
NSTEMI: Non-ST-Elevation myocardial infarction
PCWP: Pulmonary capillary wedge pressure
PVRI: Pulmonary vascular resistance index
ROC: Receiver Operating Curve
STEMI: ST-Elevation myocardial infarction
SVRI: Systemic vascular resistance index
